# Supplementary material for: Oral intake of mesoporous silica is safe and well tolerated in male humans
Source: PLoS One. 2020 Oct 2;15(10):e0240030. doi: 10.1371/journal.pone.0240030 (PMC7531782; doi:10.1371/journal.pone.0240030)
Supplement: S1 Protocol — (PDF) [file pone.0240030.s002.pdf]

Research protocol Sigrid

Tolerability and feasibility evaluation of a food additive based on porous silica compounds for weight loss and prevention of obesity related co-morbidities

Background

Obesity is a chronic disease that is difficult to treat with life style modifications only. Additional support is often required (*CN Ochner et al. Lancet 2015*). Therefore also obesity prevention is important for subjects with overweight. Safe and well tolerable food additives may be one way to improve the effect of life style modifications both for treatment of obesity and for obesity prevention in overweight subjects.

We now aim to evaluate the potential effects of silica compounds for weight loss and obesity prevention. Specific porous silica have been shown to induce weight loss in mice (*Kupferschmidt N et al. Nanomedicine 2013).* In addition, diatomaceous earth, 250mg/day has been shown to lower blood lipids in subjects with hypercholesterolemia in an open uncontrolled 8 weeks study (*Wachter H et al. Eur J Med Res 1998*). Diatomaceous earth is composed of amorphous silicates from sedimentary rock and is used as a dietary food additive for improving, for example, the shape of nails, hairs and skin. Diatomaceous silicates are approved by the US FDA as food additives.

The mechanisms inducing weight loss and the possible lipid lowering effects are still unclear. However, it is possible that the mesoporosity and large surface area porous silica materials, used in the mice study, facilitate the absorption of different biomolecules into the material. This absorption is probably both specific i.e., depending of the pore size and unspecific and more dependent of the large surface area. Gastrointestinal enzymes such as lipases have been shown to be specifically absorbed and inactivated in pores of well-defined size (*Kang Y et al. Ing End Chem Res 2007*) which will reduce the enzymatic activity and in turn may reduce the gastrointestinal uptake of nutrients.

Silica compounds have been used for oral use during several years both in the cosmetic and pharmaceutical industries. Synthetic amorphous silica are described in the U.S. Pharmacopeia and approved as food additives (E551 under EU regulations). Their use is "generally recognized as safe" (GRAS) by the FDA.

The present limit that may be safely used in food under 21 CFR 172.480 is 2% by weight of the food.

Safety aspects of silica food additives

The safety profile of silica compounds in general appears to be excellent and as mentioned above diatomaceous silicates are approved by FDA as food additive. No association between oral intake of cilica and allergy has been found. There is one case report demonstrating an association between trisilicate intake and kidney stones (Lee MH et al. Sc J Urol Nephrol 1993) but the association has been questioned in a later case report (Ichiyanagi O et al. Urology Int 1998) , The silica compounds used in this study are non-toxic, and inorganic.

The gastrointestinal uptake of the silica compound used in the mice studies, was extremely low. However, silicon from food-based sources is more readily absorbed. It has been demonstrated that food-based silica is digested and absorbed from the GI tract in humans (*Jugdaohsingh et al. Am J Clin Nutr 2002*). Silicon is mainly excreted via the urine without evidence of toxic accumulation in

the body (*Reffitt DM et al. J Inorg Biochem 1999*). A mean (± standard deviation) of 0.9 ± 36.3% of ingested silicon was excreted within 6 h after intake, with some variations depending on the silicon source, corresponding to 20 mg excreted silicon/day for a normal man (*Jugdaohsingh et al. Am J Clin Nutr 2002*). In the present study blood and urine silicon levels will be measured.

Signs of unspecific side effects as well as side effects associated with the suggested mode of action will be looked for. Unspecific local effects, such as gastrointestinal inflammation and obstipation are possible effects of large doses of silica. In the present study, inflammation markers in plasma and hemoglobin in faeces will be analyzed before and during the study period.

The mechanisms behind the weight loss properties of silica are still unclear but as mentioned above lipase inactivation is one possibility. Specific side effects of porous silica may therefore occur such as diarrhea due to reduced gastrointestinal lipase activity. In addition reduced uptake of vitamins and trace elements may also be a result of porous silica intake. In the present study blood tests for vitamins and trace elements will be performed before and during the study period. In addition, questionnaires regarding gastrointestinal symptoms and bowel emptying habits will be filled out by the study participants before and during the study.

General aim of the study:

- To study if porous silica compounds up to 9 grams per day can be safely used as food additives without significant negative side effects

Specific aims

- To study the feasibility of silica compounds in different doses and to assess whether there are dose-dependent side effects

- To determine the effects of intake of silica on gastrointestinal function and bowel emptying habits
- To study the effect of silica on circulating inflammation markers , vitamin levels and trace elements levels
- To study the effects of silica intake on blood lipids, hormone levels and body weight

Study type

Single blinded uncontrolled pilot study

Study groups

Group A healthy normal weight males n=10

Inclusion criteria

- BMI 20-25 kg/m^2
- 18-35 years old

Exclusion criteria

- Chronic somatic diseases that may affect metabolic and/or intestinal function (e.g. diabetes, hypertension, dyslipidemia, IBD, gluten intolerance, pancreatic dysfunction, other causes of malabsorption, neoplastic disease,)
- Allergies with previous anaphylactic reactions
- Previous abdominal surgery
- Current or history of eating disorders
- Extreme or unusual diets such as LCHF and vegetarian diets for the last year
- Psychiatric disorders (e.g. schizophrenia, and other diagnoses that may influence compliance)
- Drug or alcohol abuse
- Continuous oral pharmacological treatment and other types of pharmacological treatment that may influence the study
- Other conditions which the investigator considers could negatively affect the outcome of the study or study compliance

Group B obese males n=10

Inclusion criteria

- BMI 30-45 kg/m^2
- 18-35 years old

Exclusion criteria

- Chronic somatic diseases, except for obesity that may affect metabolic and/or intestinal function (e.g. diabetes, hypertension, dyslipidemia, IBD, gluten intolerance, pancreatic dysfunction, other causes of malabsorption, neoplastic disease,)
- Allergies with previous anaphylactic reactions
- Previous abdominal surgery
- Current or history of eating disorders
- Extreme or unusual diets such as LCHF and vegetarian diets for the last year Psychiatric disorders (e.g. schizophrenia, and other diagnoses that may influence compliance)
- Drug or alcohol abuse
- Continuous oral pharmacological treatment and other types of pharmacological treatment that may influence the study
- Other conditions which the investigator considers could negatively affect the outcome of the study or study compliance

Procedure

The test silica compound

- The test item, which will be used in these experiments is synthetic amorphous silica. It has the same chemical composition as approved colloidal silica (see table below). It has high

purity and it is taste and odor free. It conforms to the test requirements as published to date by the U.S. Pharmacopeia for silicon Dioxide, and meets food additive standards for E551 under EU Regulation No. 231/2012.

- A mixture of porous silica with three different pore sizes will be used. The particle size is approximately 3 micrometers. The three pore sizes will be in the range of 50-120 Å.
- The silica will be delivered in capsules containing 0.5-1.0g of the silica mixture in each capsule
- The subjects are instructed to drink a large glass (approximately 250ml) of water in connection with the intake of the capsules.

Study phase one, 21 study days

After signature on the written consent, medical examination, questions regarding eating habits, sleep patterns, living conditions and gastrointestinal health and blood and faces sampling, both the normal weight and obese subjects will receive placebo capsules study day 1-5 (five days run-in period). Thereafter all subjects will receive porous silica.

Dosage

- Study day 6-9 (four days): 1g before breakfast, lunch and dinner.
- Study day 10-14 (five days): 2g before breakfast lunch and dinner.
- Study day 15-21 (seven days,): 3g before breakfast lunch and dinner.

Dose adjustment and food intake advice

If gastrointestinal adverse events occur after increased dosage, the study staff will discuss the situation with the participant. If diarrhea occurs it might be due to the possible lipase inhibiting effect. If the participant have had a large fat intake the staff will suggest reduction of fat intake. If this has no effect or if fat intake has been low or if other gastrointestinal symptoms occur, the dose will be adjusted back to the previous one.

Examinations, time schedule

- Clinical examinations — day 1, 7, 14, 21, 28 (follow-up for normal weight participants)
- Blood sampling — day 1, 7, 14, 21
- Faeces sampling — day 1, 21
- Morning urinary sampling — day 1, 21

Phase two, extension phase, 10 study weeks

If phase one of the study is successfully completed the obese subjects will be continuously treated with the highest tolerated dose for each individual tested in phase one. The treatment will continue during 10 additional weeks, i.e., in total 12 weeks with the maximum tolerated dose.

Examinations, time schedule (after the initial three week study period in phase one)

- Clinical examinations — week 1, 5, 10, 15 (follow-up visit)
- Blood sampling — week 1, 5, 10, 15
- Faeces sampling — week 1, 10
- Morning urinary sampling — week 1, 5,10, 15

In addition, study staff will have structured and documented weekly contact with the participants by phone, e-mail or other electronic media throughout the entire study period for dose adjustments dietary advice and documentation of capsule intake and adverse events.

Clinical examinations included both in phase one and phase two:

- weight, height
- blood pressure
- cardio-respiratory and abdominal examinations.
- Questions and questionnaires regarding:
    - FFQ
    - compliance,
    - life style during the study period,
    - gastrointestinal function/habits
    - adverse events

Fasting blood analyses will include both in phase one and two:

- Routine clinical laboratory tests including
- blood cell count
- inflammation markers (CRP)
- P-glucose, lipids, insulin,
- vitamins: Retinol (vit A), 25-OH vit D
- trace elements (e.g. Mg, Zn)
- liver status
- HbA1C,
- Creatinine

In addition:

- silica analyses
- serum and plasma for storage in biobank

Faeces sampling for:

- quantification of excreted silica
- faeces hemoglobin
- calprotectin
- elastase
- lipids
- storage in biobank

Urinary sampling for

- silica concentration
